# Oral Treatment with the Pectin Fibre Obtained from Yellow Passion Fruit Peels Worsens Sepsis Outcome in Mice by Affecting the Intestinal Barrier

**DOI:** 10.3390/ph17070863

**Published:** 2024-07-01

**Authors:** Bruna C. da Silveira, Fernanda da Silva Platner, Liza B. da Rosa, Matheus L. C. Silva, Karien S. da Silva, Natalia M. T. de Oliveira, Eduardo B. Moffa, Karinny F. Silva, Lídio G. Lima-Neto, Daniele Maria-Ferreira, Lucimara M. C. Cordeiro, Marcelo B. Gois, Elizabeth S. Fernandes

**Affiliations:** 1 Programa de Pós-Graduação em Biotecnologia Aplicada à Saúde da Criança e do Adolescente, Faculdades Pequeno Príncipe, Curitiba 80230-020, PR, Brazil; brunac_silveira@yahoo.com.br (B.C.d.S.); fsilvaplatner@gmail.com (F.d.S.P.); lizabdarosa@gmail.com (L.B.d.R.); lintz.matheus@gmail.com (M.L.C.S.); karien.sauruk@pelepequenoprincipe.org.br (K.S.d.S.); natimulinari@gmail.com (N.M.T.d.O.); danielemariaferreira@gmail.com (D.M.-F.); 2 Instituto de Pesquisa Pelé Pequeno Príncipe, Curitiba 80250-060, PR, Brazil; 3 College of Dentistry, University of Saskatchewan, Saskatoon, SK S7N 5E5, Canada; eduardo.moffa@usask.ca; 4 Programa de Pós-Graduação, Universidade Ceuma, São Luís 65075-120, MA, Brazil; karinnyfarias20@gmail.com (K.F.S.); lidio.neto@ceuma.br (L.G.L.-N.); 5 Departmento de Bioquímica e Biologia Molecular, Universidade Federal do Paraná, Curitiba 81531-980, PR, Brazil; lucimaramcc@ufpr.br; 6 Faculdade de Ciências da Saúde, Universidade Federal de Rondonópolis, Rondonópolis 78740-393, MT, Brazil; marcelobiondaro@gmail.com

**Keywords:** soluble dietary fibres, pectin, sepsis

## Abstract

The biological activities of plant-derived soluble dietary fibres (SDFs) have been widely investigated. Pectin from yellow passion fruit (YPF-peSDF) peels was suggested as a protective macromolecule in ulcers and colitis due to its antioxidant and anti-inflammatory properties. Sepsis has high mortality and morbidity and is characterised by inflammatory and oxidative stress imbalances. Evidence suggests that pectins may aid sepsis treatment; however, the effects of YPF-peSDF on sepsis remain unclear. Herein, polymicrobial sepsis was induced by cecal-ligation and puncture in mice treated with YPF-peSDF (1 and 10 mg/kg; gavage). YPF-peSDF accelerated mortality, reaching 100% in 24 h. Inflammation was present in the colons and small intestines (SI) of both vehicle- and fibre-treated mice. Although crypt depth and width, and villus height were preserved in the SI of septic mice administered YPF-peSDF, they exhibited exacerbated muscle layer atrophy and mucosa and submucosa hypertrophy, along with shortened enterocytes. Larger crypts and shorter enterocytes were noted in their colons in comparison with vehicle-controls. YPF-peSDF also reduced inflammatory cell numbers and exacerbated IL-6 levels in peritoneal lavage fluid (PELF) samples. YPF-peSDF modulated SI but not colon cytokines. Lipoperoxidation and antioxidant capacity levels were attenuated in PELF samples. Overall, in contrast to previous evidence, YPF-peSDF worsened polymicrobial sepsis outcomes in mice.

## 1. Introduction

Dietary fibres are carbohydrates widely found in plants and other natural sources, including seaweeds, that are classified as soluble (SDFs) or insoluble in water (IDFs). The biological activities of SDFs from different plant parts have been broadly investigated, with those isolated from agriculture wastes being particularly interesting. A range of carbohydrates has been detected in SDFs, and their activities may vary according to their composition, physicochemical properties, and sources. Examples of SDF biological activities include the modulation of inflammation and oxidative stress [1,2,3,4,5].

Pectin is a structural cell carbohydrate polymer of high-molecular-weight (50–150 kDa) found in all plants, commonly used in the food and beverage industry, due to its ability to increase viscosity and bind water. It presents a linear structure composed of galacturonic acid monomer units linked via glycosidic bonds, forming a backbone structure that can be replaced by rhamnopyranose units with chains of galactose, mannose, glucose, and xylose [6]. Methyl esterification of galacturonic acid occurs in pectin, forming high (>50% esterified galacturonic acid residues) or low methoxyl (less than 50% esterified galacturonic acid residues) pectin [6]. The beneficial effects of pectin from various sources have been described in different diseases in which inflammation and oxidative stress play a determinant role [1,7,8,9,10,11,12,13].

Sepsis is a “life-threatening organ dysfunction caused by a deregulated host response to infection” [14], which affects, at higher rates, the elderly, pregnant or recently pregnant women, neonates, hospitalised and intensive care unit patients, immunocompromised individuals, and those with chronic medical conditions [15,16,17]. It can be caused by various micro-organisms and affect millions of people worldwide, with high morbidity and mortality rates [17]. A deregulated inflammatory state (hyperinflammatory response followed by immunosuppression) is a hallmark of this condition [18], which remains poorly treated, mainly due to antimicrobial resistance (especially by bacteria; [19,20]), and inefficient modulation of inflammatory responses [15,18].

Interestingly, pectins from different plants were shown to protect against sepsis in rodents [21,22,23,24] and patients [25]; indicating the potential of these fibres to modulate infectious diseases. Recent studies isolated and characterised the pectin fibre obtained from yellow passion fruit (YPF-peSDF) peels [26], and demonstrated its ability to protect rodents from gastric ulcerative lesions and colitis; its actions were attributed to antioxidant and anti-inflammatory properties [1,13]. However, the therapeutic potential of YPF-peSDF in sepsis remains unclear. Herein, we investigated the effects of YPF-peSDF in a mouse model of polymicrobial sepsis induced by cecal-ligation and puncture (CLP). We found that YPF-peSDF administration alters the intestinal histoarchitecture and favours a deleterious phenotype in septic mice.

## 2. Results

### 2.1. YPF-peSDF Accelerates Mortality and Enhances Hypothermia in Septic Mice

**Figure 1:**
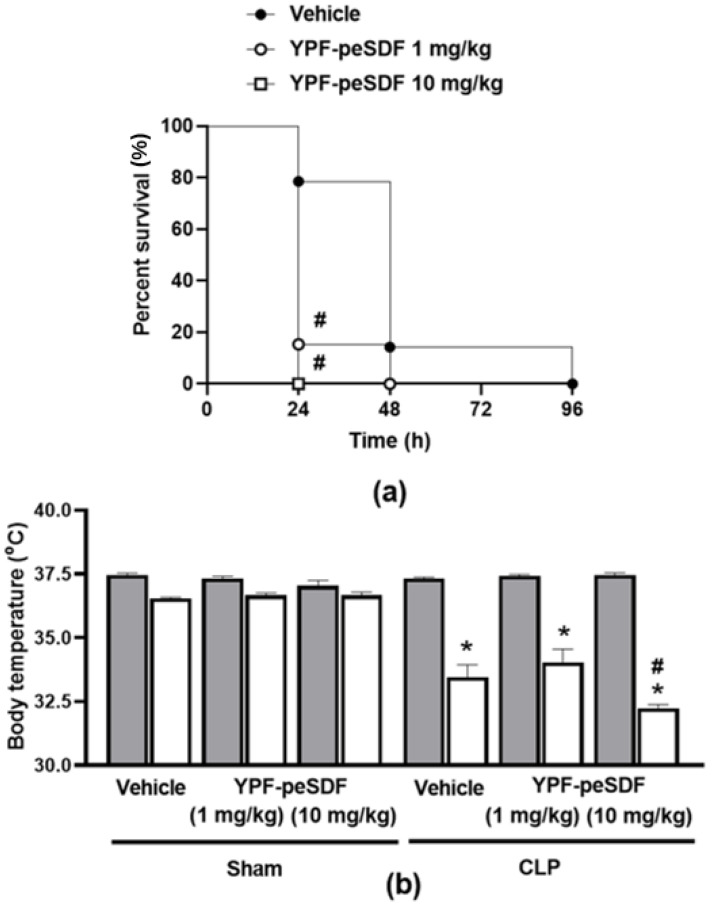
YPF-peSDF effects on mortality and body temperature. (**a**) Percent survival over 96 h in animals (n = 14; 7 males and 7 females/group) treated with either YPF-peSDF (1 and 10 mg/kg) or vehicle (saline; 5 mL/kg) by gavage 1 h prior to sepsis induction by cecal-ligation and puncture (CLP). (**b**) Body temperature measurements were taken at baseline conditions (grey bars) and 18–24 h (premortality endpoint; white bars) following surgery from Sham and CLP mice (n = 12; 6 males and 6 females/group) treated with YPF-peSDF (1 and 10 mg/kg) or vehicle (saline; 5 mL/kg) by gavage 1 h prior to surgery. * *p* < 0.05, differs from baseline; ^#^ *p* < 0.05, differs from vehicle-treated CLP mice.

Figure 1a,b demonstrates that treatment with YPF-peSDF (1 and 10 mg/kg) increases fatality and hypothermia in septic mice in comparison with vehicle (saline)-treated controls. Mortality for those that received YPF-peSDF was between 80 and 100% in the first 24 h post-sepsis induction versus 21% for vehicle (saline)-treated animals. The highest mortality rate was observed for 10 mg/kg YPF-peSDF. Although all mice presented hypothermia, 10 mg/kg YPF-peSDF-treated animals had the greatest drop in body temperature (>5.0 °C). As both doses of YPF-peSDF presented similar deleterious actions on septic mice, the dose of 10 mg/kg was chosen for the subsequent analyses.

### 2.2. YPF-peSDF Alters the Small Intestine and Colon Histoarchitectures in Septic Mice

**Figure 2:**
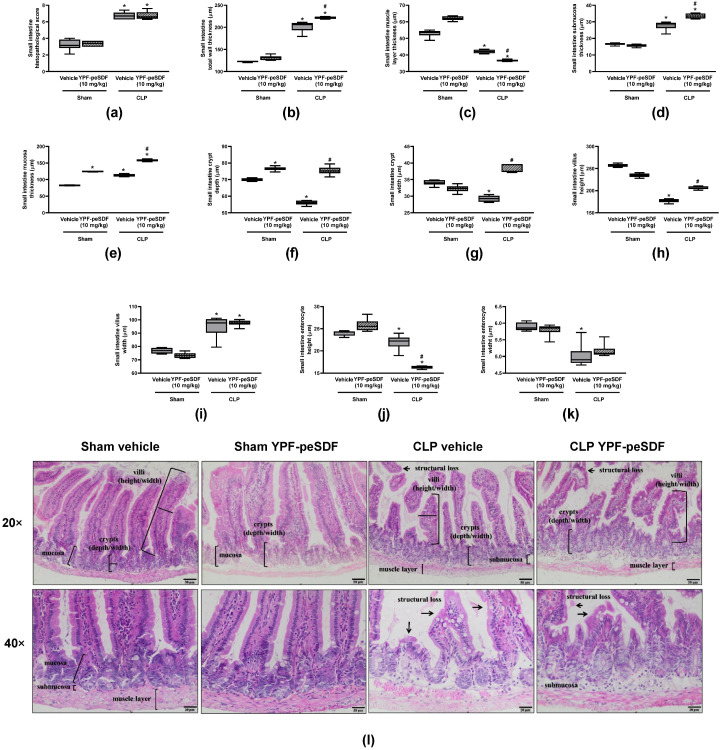
YPF-peSDF effects on small intestine histology. Histological analysis of small intestine samples obtained from Sham and cecal-ligation and puncture (CLP) mice (n = 6; 3 males and 3 females/group) treated with YPF-peSDF (10 mg/kg) or vehicle (saline; 5 mL/kg) 1 h prior to surgery. Small intestine (**a**) histopathological score, (**b**) total wall, (**c**) muscle layer, (**d**) submucosa and (**e**) mucosa thickness, crypt (**f**) depth and (**g**) width, villus (**h**) height and (**i**) width, enterocyte (**j**) height, and (**k**) width. Representative panels of the histological analysis, and (**l**) representative panels of histological analysis (20 and 40× magnification). Arrows indicate structural tissue loss, and brackets indicate specific intestinal structures. * *p* < 0.05; differs from vehicle-treated Sham animals. ^#^ *p* < 0.05; differs from vehicle-treated CLP mice.

Figure 2 depicts the alterations caused by CLP in the small intestine in comparison with control (Sham) mice. A range of significant morphological changes were noted in septic mice, including increased submucosa and mucosa layer thickness; atrophy of the muscle layer; reductions of crypt depth and width, villus height; shortening of enterocytes; and enlargement of villus width (Figure 2a–l). Although no differences were noted in the overall histopathological scores between groups (Figure 2a), YPF-peSDF (10 mg/kg) exacerbated the thickness alterations of the muscle, submucosa, and mucosa layers (Figure 2c–e), resulting in greater total wall thickness in septic mice (Figure 2b). Fibre administration in CLP animals also resulted in further shortening of the enterocytes (Figure 2j) and increased crypt depth and width, and villus height (Figure 2f–h). No effects were observed for YPF-peSDF on septic small intestine villus and enterocyte widths in comparison with the vehicle group (Figure 2i,k). In Sham mice, YPF-peSDF augmented the mucosa thickness and crypt depth (Figure 2e,f). Representative panels of the small intestine are seen in Figure 2l.

**Figure 3:**
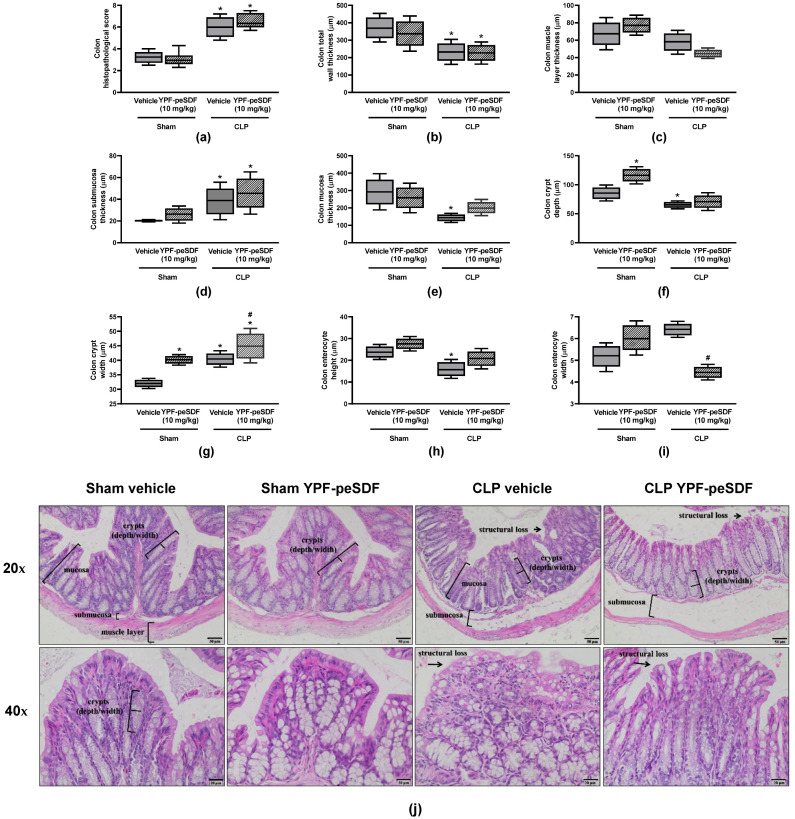
YPF-peSDF effects on colonic histology. Histological analysis of colon samples obtained from Sham and cecal-ligation and puncture (CLP) mice (n = 6; 3 males and 3 females/group) treated with YPF-peSDF (10 mg/kg) or vehicle (saline; 5 mL/kg) 1 h prior to surgery. Colonic (**a**) histopathological score, (**b**) total wall, (**c**) muscle layer, (**d**) submucosa and (**e**) mucosa thickness, crypt (**f**) depth and (**g**) width, enterocyte (**h**) height and (**i**) width, and (**j**) representative panels of histological analysis (20 and 40× magnification). Arrows indicate structural tissue loss, and brackets indicate specific intestinal structures. * *p* < 0.05; differs from vehicle-treated Sham animals. ^#^ *p* < 0.05; differs from vehicle-treated CLP mice.

Figure 3 contains the colonic histological features of Sham and CLP mice treated with either vehicle or YPF-peSDF (10 mg/kg). Sepsis promoted marked structural colon changes (Figure 3a–j) such as: hypertrophy of the submucosa layer; reductions of the mucosa layer, total wall thickness, crypt depth, and enterocyte height; and increased crypt width. Treatment with YPF-peSDF further augmented crypt width (Figure 3g) while attenuating that of enterocytes (Figure 3i) in septic mice. Marked alterations (greater crypt depth and width) were also observed in Sham mice administered with YPF-peSDF (Figure 3f,g). Representative panels of the colons are seen in Figure 3j.

### 2.3. YPF-peSDF Diminishes Inflammatory Cell Influx into the Peritoneum without Affecting Cell Accumulation and Cryptitis in the Intestine of Septic Mice

A significant increase in the number of inflammatory cells was observed in PELF, small intestine, and colon samples of CLP in comparison with Sham animals (Table 1). A similar effect was noted for the development of cryptitis (Table 1). YPF-peSDF (10 mg/kg) did not affect leukocyte influx or cryptitis scores in the small intestine or colon, but it reduced the number of both mononuclear (52%) and polymorphonuclear (45%) cells in the peritoneum (Table 1).

### 2.4. Cytokine Release and Oxidative Stress Are Modulated by YPF-peSDF in Septic Mice

**Figure 4:**
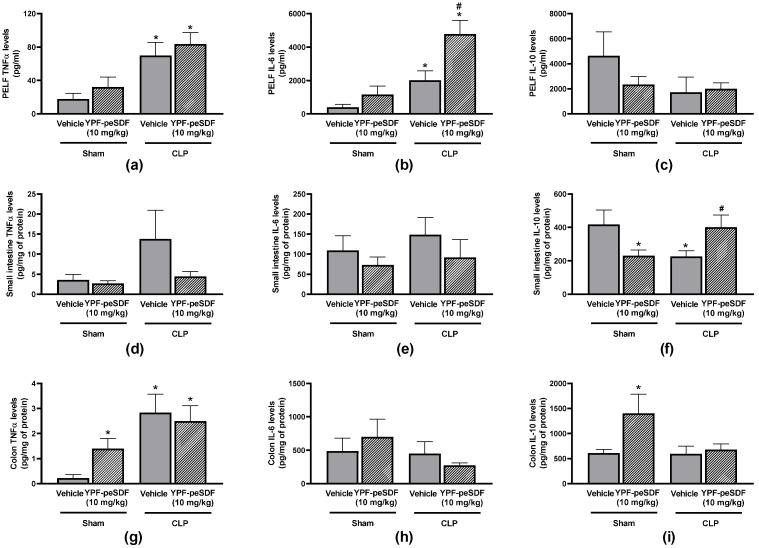
YPF-peSDF effects on cytokine generation. Peritoneal lavage fluid (PELF), small intestine, and colon samples were collected at the premortality endpoint (18–24 h) from Sham (n = 10; 5 males and 5 females/group) and cecal-ligation and puncture (CLP) mice (n = 12; 6 males and 6 females/group) treated with YPF-peSDF (10 mg/kg) or vehicle (saline; 5 mL/kg) 1 h prior to surgery. Levels of PELF (**a**) TNFα, (**b**) IL-6, and (**c**) IL-10; small intestine (**d**) TNFα, (**e**) IL-6, and (**f**) IL-10; and colon (**g**) TNFα, (**h**) IL-6, and (**i**) IL-10. * *p* < 0.05; differs from vehicle-treated Sham animals. ^#^ *p* < 0.05; differs from vehicle-treated CLP mice.

Figure 4a–i depicts the effects of YPF-peSDF (10 mg/kg) on the production of PELF and intestinal tissue cytokines. As expected, sepsis promoted a significant increase in PELF TNFα (4-fold) and IL-6 (5.1-fold) production (Figure 4a–c). TNFα levels were also enhanced in the small intestines (3.9-fold) and colons (12.7-fold) of septic mice (Figure 4d,g). On the other hand, IL-10 secretion was diminished (46%) in the small intestines of the same mice in comparison with vehicle-treated Shams (Figure 4f). Septic mice treated with YPF-peSDF (10 mg/kg) presented with higher production of PELF IL-6 and small intestine IL-10, and lower quantities of TNFα in their small intestines (Figure 4a,d,f). PELF TNFα (Figure 4a), IL-10 (Figure 4c), small intestine IL-6 (Figure 4e), and colon cytokines (Figure 4g–i) of septic mice were unaffected by YPF-peSDF. Conversely, the polysaccharide administration to Sham animals induced the generation of both TNFα (6.4-fold) and IL-10 (2.3-fold) in their colons (Figure 4g,i) and diminished small intestine IL-10 (45%; Figure 4f).

**Figure 5:**
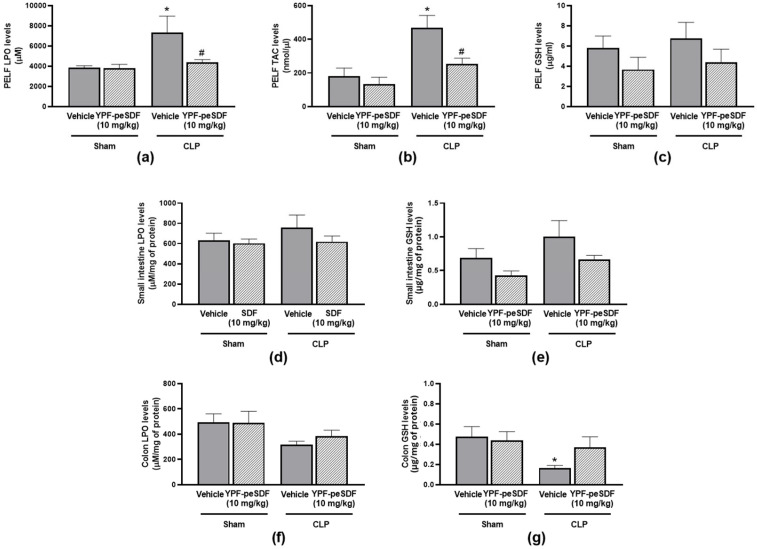
YPF-peSDF effects on oxidative stress. Peritoneal lavage fluid (PELF), small intestine, and colon samples were collected at the premortality endpoint (18–24 h) from Sham (n = 10; 5 males and 5 females/group) and cecal-ligation and puncture (CLP) mice (n = 12; 6 males and 6 females/group) treated with YPF-peSDF (10 mg/kg) or vehicle (saline; 5 mL/kg) 1 h prior to surgery. Levels of PELF (**a**) LPO, (**b**) TAC, and (**c**) GSH; small intestine (**d**) LPO, and (**e**) GSH, and colon (**f**) LPO and (**g**) GSH. * *p* < 0.05; differs from vehicle-treated Sham animals. ^#^ *p* < 0.05; differs from vehicle-treated CLP mice.

Sepsis-induced rises of LPO (1.9-fold) and TAC (2.6-fold) levels in the peritoneum (Figure 5a,b) without altering GSH production (Figure 5c). No differences were observed between groups in regards to small intestine LPO and GSH levels or colon LPO (Figure 5d–f). On the contrary, colon GSH was reduced in septic mice treated with vehicle when compared with their Sham counterparts. YPF-peSDF (10 mg/kg) markedly reduced both PELF LPO (40%) and TAC (46%) levels, with no effects on peritoneal GSH (Figure 5a–c). Similarly, the polysaccharide had no effects on small intestine or colon GSH and LPO levels (Figure 5d–g).

## 3. Discussion

Sepsis is a harmful condition that can evolve into a lethal outcome and cause great morbidity in those who survive the infection. Sepsis treatment is primarily performed through antibiotic administration, which frequently fails due to micro-organism resistance, late diagnosis, and/or medical assistance. In this context, novel therapies able to treat or aid sepsis treatment are essential to better clinical management.

Plant-derived pectins have been shown to protect against sepsis in rodents [21,22,23,24] and patients [25]; indicating their potential to modulate responses to infectious diseases. The yellow passion fruit (*Passiflora edulis* f. *flavicarpa*) is native to Brazil and has several already defined biological activities for the plant, including sedative [27], anti-anxiety [27,28], antihypertensive [26], antitumour [29], hypoglycaemic [30], anti-obesogenic [31], and nephroprotective [32]; amongst others. The digestive tract is frequently affected during sepsis, and there is growing evidence that the intestines can modulate a series of diseases [33,34]. Additionally, the advantageous effects of fibre consumption on intestinal health have been well documented in the last few years [35,36].

Recent studies demonstrated the ability of yellow passion fruit-soluble dietary fibres (rich in pectin), denominated YPF-peSDF herein, to protect the intestinal and gastric mucosae [1,13,37]. An initial study evaluated the effects of different oral doses of YPF-peSDF in a rat model of acute gastric damage caused by ethanol intake [13]. The fibres were given 1 h prior to lesion induction and displayed gastroprotective actions, preventing mucus depletion and oxidative stress; these, were dose-independent. Additionally, protection of the intestinal barrier was observed in mice damaged by dextran sodium sulphate-induced colitis treated with YPF-peSDF for two-days after disease induction; an effect suggested to be due to decreased tissue inflammation [37]. Similarly, mice that received repeated oral treatments with YPF-peSDF prior to 5-fluorouracil-induced mucositis, exhibited preserved intestinal structures due to the prevention of tissue inflammation and oxidative stress [1].

Herein, despite the YPF-peSDF ability to modulate inflammation (by elevating PELF IL-6 and small intestine IL-10 and reducing small intestine TNFα generation and leukocyte accumulation in the peritoneum) and oxidative stress (by attenuating PELF LPO and TAC levels), the fibre was not able to protect against sepsis-induced mortality and hypothermia and, on the contrary, accelerated mortality and exacerbated hypothermia in septic mice. This was surprising considering that the above-discussed reports were developed with similar doses of YPF-peSDF to those evaluated in the present study and were associated with lower disease morbidity. This evidence and the previous data showing that the ingestion of this fibre can preserve intestinal tissue homeostasis in damaging settings [1,37], led us to investigate the effects of YPF-peSDF on the intestinal histoarchitecture.

It was found that animals with sepsis pretreated by oral route with YPF-peSDF (10 mg/kg) present with important small intestine and colon structural changes in comparison with vehicle-administered controls. YPF-peSDF aggravated the alterations seen in the small intestine muscle, submucosa, and mucosa layers of septic mice, leading to greater shortening of enterocytes and increases in crypt depth and width, and villus height. YPF-peSDF also further increased crypt width while reducing that of enterocytes in septic mice. YPF-peSDF also promoted important alterations in Sham mice, including thickening of the small intestine mucosa and increased crypt depth, in addition to significant changes (greater crypt depth and width) in their colons. Although a regulatory effect was noted for the fibre in the generation of cytokines in the small intestine, YPF-peSDF had no effects on the cryptitis and leukocyte influx in the intestinal tissue of septic mice. All these alterations caused by YPF-peSDF may impact intestinal movement, nutrient, water, vitamin, and ion uptake, as well as tolerance to microbes, amongst other intestinal functions. Indeed, disruption of the intestinal barrier has been associated with worsening sepsis outcomes once it facilitates tissue permeability to pathogens and their antigens and their subsequent spread to the circulation [38,39,40,41].

This collection of data demonstrates that YPF-pe-SDF is deleterious to septic conditions and is contrary to the evidence that previously showed that these fibres protect against intestinal damage in models of chemotherapy-induced mucositis and dextran sodium sulphate-induced colitis [1,37]. Although these are all models characterised by severe intestinal inflammation, it is possible that the discrepancies between our results and those obtained in the other models are due to the nature of the injury (i.e., acute intestinal injury caused by CLP versus a slower disruption of the intestinal barrier caused by colitis or mucositis). While doses do not seem to influence the outcome, it is important to highlight the possible impact of the frequency of treatment (repeated versus single treatment) as well as its start (prior to versus after disease development).

## 4. Materials and Methods

### 4.1. Animals

Inbred male and female C57BL/6 mice (8 weeks old) were obtained from the Fundação Oswaldo Cruz-FIOCRUZ (Paraná, Brazil) following approval of the experimental procedures by the Animal Use Ethics Committee of the Instituto de Pesquisa Pelé Pequeno Príncipe (IPPPP), under the protocol number 050/2020. Animals were housed at the Biological Service Unit of IPPPP for 3 weeks before the experiments. All mice were housed under a 12-h light/dark cycle, at a controlled environmental temperature (21 ± 2 °C) and humidity (60 ± 5%). All the experimental groups were matched for gender and body weight. All experiments followed the recommendations of the Brazilian guidelines on animal experimentation of the National Council for the Control of Animal Experimentation (CONCEA) and the ARRIVE guidelines [42,43].

### 4.2. Sepsis Induction

A total of 130 mice were used in the study (65 males and 65 females). Except for mortality (observation period of 96 h), experiments were designed with a premortality endpoint (between 18 and 24 h; based on body temperature measures; heat loss of 10–15%). Mortality experiments were performed with 21 male (n = 7/group) and 21 female (n = 7/group) mice.

Sepsis was induced in animals after they received a single intramuscular ipsilateral injection containing midazolam (2 mg/kg; Dormire^®^, Cristália, Brazil), 15 min prior to anaesthesia induction with a mixture of ketamine (50 mg/kg) and xylazine (1 mg/kg) via i.m. injection (contralateral side). Surgery was performed using aseptic techniques. An incision of ~2 cm was created in the shaved ventral surface of the abdomen, and the cecum was exposed through the incision. The cecum was ligated at its base (without causing bowel obstruction) with silk 4-0 suture (Shalon Medical; São Luís de Montes Belos, GO, Brazil) and perforated with a 22-gauge needle, resulting in two holes. Sham-operated animals were used as controls. Both sham and CLP mice were sutured with absorbable sutures (Vicryl; Shalon Medical) and received 1 mL of saline 0.9% (subcutaneously; Samtec Biotecnologia; Ribeirão Preto, SP, Brazil) for post-surgery hydration. Body temperature was measured at baseline and between 18 and 24 h after surgery. Then, animals were killed by anaesthesia with ketamine and xylazine, followed by a cardiac puncture, and samples were collected. A laparotomy was performed [44], the peritoneal cavity was washed with 2.0 mL of sterile phosphate buffered-saline (PBS; Sigma-Aldrich; São Paulo, SP, Brazil), and the peritoneal exudate lavage fluid (PELF) was collected for further analysis of cell counts, cytokines, and oxidative stress markers. The small intestines and colons were collected for histology and measurement of cytokines and oxidative stress markers.

### 4.3. Treatment

YPF-peSDF is a pectin composed mainly of homogalacturonan (92% galacturonic acid) with a high content of methyl esters (~70%), previously characterised by Abboud and collaborators [13]. The fibre was obtained as previously described [13] by the sequential enzymatic digestion of the fruit peel flour by heat-stable α-amylase, protease, and amyloglucosidase.

In the mortality experiments, mice received either vehicle (sterile phosphate buffered-saline; PBS; 5 mL/kg) or YPF-peSDF (1 and 10 mg/kg [13]), per os (p.o.), 1 h prior to and at every 24 h post-surgery. For sample collection, animals were treated with a single administration of the pectin fibre (10 mg/kg; p.o.), 1 h prior to surgery.

### 4.4. Small Intestine and Colon Histological Analysis

#### 4.4.1. Sample Collection and Preparation

Small intestine and colon samples (n = 6/group) were collected from animals at premortality endpoint. Samples were placed in buffered formalin (Neon Comercial; Suzano, SP, Brazil) for 16 h and then, transferred to ethanol and ether solutions (Abba Quimica; Curitiba, PR, Brazil). Samples were embedded in paraffin, and 5 µm sections were cut and used for histology. The sections were stained with hematoxylin and eosin (H&E). Images (20 and 40× magnification) were captured in a digital camera (Olympus^®^ (Tokyo, Japan) CX43RF, 3.0 megapixel) connected to an optical microscope (Olympus^®^ EP50) and used for analysis (4 quadrants/image/mouse; n = 6 mice/group) of histopathology and tissue morphometry.

#### 4.4.2. Histopathology and Morphometry

Small intestine and colon sections (n = 6/group) stained with H&E were analysed by the following parameters scored from 0–3 in comparison with normal tissues (0 = not present; 3 = severe alteration): (i) presence/distribution of inflammatory cells in the submucosa or intestinal mucosa, (ii) loss of the architecture of the intestinal mucosa denoted by flattening of the mucosa, depletion of goblet cells, epithelial erosion, ulceration, or abscess formation, and (iii) inflammatory infiltrates in the intestinal crypts. Images were examined by a blind investigator, under light microscopy (Olympus^®^ BX43F; Tokyo, Japan) with 40–100× objectives [1].

The same images were analysed under light microscopy (Olympus^®^ BX43F; Tokyo, Japan) with 10× objective, for the following morphometric parameters: thickness of the muscular, sub-mucosa, and mucosa layers, the depth and width of the crypts, and the height and width of the enterocytes, by using a high-resolution camera (Olympus^®^ SC30, 3.0 MP). Quantification of each parameter was performed using the Image-Pro Plus software (version 4.5.0.29; BioImager; Richmond Hill, ON, Canada; [45]).

### 4.5. Total Antioxidant Capacity

Total antioxidant capacity (TAC) was measured in PELF samples by using a commercial kit (Total Antioxidant Capacity Assay Kit; Sigma-Aldrich; São Paulo, SP, Brazil). Briefly, samples (100 µL/per well; 1:10) were incubated with a 100-µL Cu^+2^ solution for 90 min, at room temperature in the dark. The absorbances were read at 570 nm, and compared with a standard curve of trolox (0–20 nM/well), a water-soluble vitamin E analogue. The results are expressed as nmole/microlitre (nmol/µL) of sample.

### 4.6. Lipid Peroxidation

Lipid peroxidation (LPO) was measured in PELF, small intestine, and colon samples as previously described by Jiang et al. [46], modified. PELF and tissue homogenates (prepared in PBS containing protease inhibitors; SigmaFastTM; Sigma-Aldrich; São Paulo, SP, Brazil) (300 µL) were vortexed with 30 μL 90% methanol in 2.0 mL tubes, and then, centrifuged at 2800 rpm, 4 °C, for 10 min. The resulting supernatant (60 μL) was transferred to a new tube and incubated with 600 μL of ferrous oxidation-xylenol orange reagent (FOX; composed by: butylated hydroxytoluene (4 mM; Labsynth; Diadema, SP, Brazil), ferrous sulphate (FeSO_4_, 250 mM; Labsynth; Diadema, SP, Brazil), sulfuric acid (H_2_SO_4_, 25 mM; Exodo Cientifica; Sao Paulo, SP, Brazil), and orange xylenol (100 mM; Neon Comercial; Sao Paulo, SP, Brazil)), in the dark, at room temperature for 30 min. Then, the samples were centrifuged at 2800 rpm, 4 °C, 10 min, and the supernatants were used for the assay. Samples (300 µL) were added per well, and the absorbances were read at 560 nm and compared with a hydrogen peroxide (H_2_O_2_) standard curve (0–100 µM). A blank well-containing methanol (1:10 in FOX reagent) was used and subtracted from the samples for calculation. The results are expressed in micromolar (µM; PELF) or µM/milligram (mg) of protein (tissue samples). Protein from tissue proteins was measured as per the manufacturer’s instructions (QuantiPro™ BCA Assay Kit; Sigma-Aldrich; São Paulo, SP, Brazil).

### 4.7. Reduced Glutathione Tissue Levels

Reduced glutathione (GSH) levels were measured in PELF, small intestine, and colon samples. For this, tissues were homogenised in PBS containing protease inhibitors (SigmaFastTM; Sigma-Aldrich; São Paulo, SP, Brazil), and centrifuged at 8900 rpm, 4 °C, for 20 min. Then, 40 µL of each sample were precipitated with 50 40 µL 12.5% trichloroacetic acid and centrifuged at 3000 rpm, 4 °C, 15 min. In a 96-well plate, 10 µL of the tissue supernatants and PELF samples were added to 290 µL of TRIS-HCl buffer (400 mM; pH 8.5; Sigma-Aldrich; São Paulo, SP, Brazil) and 5 µL 10 mM 5,5′-dithiobis-(2-nitrobenzoic acid) (DTNB; Sigma-Aldrich; São Paulo, SP, Brazil). The absorbances were then measured at 420 nm and compared to a GSH standard curve (0–400 μg/mL). The results are expressed as microgram/millilitre (µg/mL; PELF) or microgram/milligram (µg/mg) of protein (tissue samples). Protein from tissue proteins was measured as per the manufacturer´s instructions (QuantiPro™ BCA Assay Kit; Sigma-Aldrich; São Paulo, SP, Brazil).

### 4.8. Cytokine Measurements

Cytokines (TNFα, IL-6, and IL-10) levels were measured in PELF, small intestine, and colon samples. Tissues were homogenised in PBS containing protease inhibitors (SigmaFastTM; Sigma-Aldrich; Brazil), centrifuged at 8900 rpm, 4 °C, for 20 min, and the supernatants used for the assay, performed according to the manufacturer´s instructions (Peprotech; Ribeirão Preto, SP, Brazil), by using commercial kits. The results are expressed as picogram/millilitre (pg/mL; PELF) or picogram/milligram (pg/mg) of protein (tissue samples). Protein from tissue proteins was measured as per the manufacturer´s instructions (QuantiPro™ BCA Assay Kit; Sigma-Aldrich; São Paulo, SP, Brazil).

### 4.9. Statistical Analyses

The results are presented as mean ± mean standard error (SEM) or median (minimum-maximum). For multiple statistical comparisons between groups, data were analysed by two-way or one-way analysis of variance (ANOVA), followed by the Bonferroni test with FDR correction. Paired t tests were used when appropriate. Histology scores were analysed using Kruskal–Wallis test, followed by Dunn’s test for multiple comparisons. All data were analysed in GraphPad Prism 8.0. (now Dotmatics; Woburn, MA, USA); *p* < 0.05 was considered significant. All n numbers are indicated on the graphs.

## 5. Conclusions

Homeostasis of the intestinal barrier and function is essential to health maintenance. Sepsis may originate from an infection, starting as a result of an intestinal, injury following mechanical trauma or even chronic diseases such as colitis and mucositis. Therapies that contribute to intestinal integrity while protecting against sepsis-induced inflammation and oxidative stress may represent interesting approaches to the clinical management of this syndrome. Herein, we demonstrated that YPF-peSDF, previously shown as protective against intestinal damage in rodents with colitis and mucositis, is actually, deleterious in sepsis, accelerating mortality and exacerbating hypothermia. This effect was associated with important changes in the small intestine and colon structures, as well as in peritoneal and small intestine inflammation and oxidative stress. Overall, the obtained evidence indicates that the type of intestinal injury and treatment schedules may influence the effects of this fibre. Nonetheless, further studies on the YPF-peSDF biological activities deserve attention, considering its importance as an agricultural waste of yellow passion fruit peels.

## Figures and Tables

**Table 1:** Effects of YPF-peSDF on peritoneal, small intestine, and colon inflammation. Samples were collected at the premortality endpoint from Sham and cecal-ligation and puncture (CLP) animals treated with either YPF-peSDF (10 mg/kg) or vehicle (saline 5 mL/kg), 1 h prior to surgery.

	Parameters	Sham Vehicle	Sham YPF-peSDF	CLP Vehicle	CLP YPF-peSDF
PELF inflammatory cells	Total cells (×10^6^)	2.97 ± 0.9	3.24 ± 1.0	6.61 ± 1.7 *	2.98 ± 0.5 ^#^
	PMN cells (×10^6^)	1.37 ± 0.4	1.30 ± 0.4	2.54 ± 0.6 *	1.41 ± 0.3 ^#^
	Mononuclear cells (×10^6^)	1.61 ± 0.5	1.94 ± 0.6	4.10 ± 1.2 *	1.97 ± 0.4 ^#^
Small intestine leukocyte influx	Score (median (min–max))	1.1 (0.7–1.4)	1.2 (0.7–1.3)	2.1 (1.5–2.6) *	2.2 (2.0–2.5) *
Colon leukocyte influx	Score (median (min–max))	1.1 (0.6–1.2)	1.0 (0.7–1.4)	2.0 (1.1–2.5) *	2.1 (1.7–2.6) *
Cryptitis	Score (median (min–max))	1.0 (0.5–1.5)	1.1 (0.6–1.4)	2.5 (2.0–2.8) *	2.3 (2.0–2.8) *

* *p* < 0.05; differs from vehicle-treated Sham animals. ^#^ *p* < 0.05; differs from vehicle-treated CLP mice.

## Data Availability

Data is contained within the article.
